# Lithotrity and Dilatation of the Urethra for Stone in the Female Bladder

**Published:** 1830-01-01

**Authors:** 


### LITHOTRITY AND DILATATION OF THE URETHRA, FOR STONE IN THE FEMALE BLADDER.*

The following case is particularly valuable at the present time when the question of lithotrity is *boring* some good folks' brains very sadly. The Matty Marvellous's of the profession will be amazed to find that the new method can prove, under any circumstances, less expeditious or more difficult than the old one, but nevertheless the fact is so. We have reason to know that an eminent, we may say the most eminent, surgeon in this good city was foiled in lithotriting a stone in the female bladder not a hundred years ago. But to the present case; which is recorded by Mr. James Wilson.

"Mrs. H., æt. 68, had been subject to calculous complaints for 20 years, and had passed many small stones; for several months she had voided none, and her sufferings were become greatly aggravated. On introducing a sound a large stone was easily felt. I was in-

* Glasg. Journ. No. VIII.

duced, in this case, to try lithotrity; 1st, on account of the very favourable reports given of that operation; and, 2dly, because the stone in this case appearing to be very large, it was probable that by breaking it down, the fragments might be easily removed by dilatation of the urethra. I was aware that in two cases in which dilatation of the urethra had been tried here, the stones, from their very large size, required a great deal of force for their removal; this, doubtless, was the cause of incontinence of urine, which continued in both cases for a considerable time.

"On the 8th of August, after filling the bladder with warm water, Civiale's instrument, *à trois branches*, made by Weiss, was easily introduced, and placed in contact with the stone. It was found, however, that the bladder could not be kept distended, the injected fluid escaping by the sides, and also through the centre of the instrument, which I have no doubt added both to the difficulty and danger of the operation. Some of the lithotrity instruments are so constructed, that without their removal, the bladder may be distended by injection. The want of such an apparatus in the instrument which I employed, was found to be a serious defect. Many unsuccessful attempts were made to grasp the stone, and there was good reason to think, that when the bladder became empty on the escape of the fluid, and contracted round the stone, its coats became entangled in the claws of the instrument. Much irritation was occasioned, and a considerable discharge of blood took place. At length, by raising the patient, who till now lay in a horizontal position, to a semi-sitting posture, the stone was partially seized, and drilled to the extent of a quarter of an inch. It then became necessary to change the position of the stone, in order to present a new surface for trituration, but it could not again be laid hold of, and the instrument was withdrawn, after continuing the attempts for at least three quarters of an hour. The patient was a good deal exhausted. Sixty drops of tinct. opii were given, and strict antiphlogistic treatment enjoined. No feverishness followed, and the pain, though severe, was certainly less than might have been expected from the irritation produced by this operation. Some days afterwards, the paroxysms of pain occasioned by the stone became very frequent, and so severe, that on the 18th, ten days after the attempt at lithotrity, it was deemed necessary to do something for the removal of the calculus. From the total failure of the former operation, it would have been wrong to have subjected the patient to a repetition of the same risk, without the probability of removing the stone, which there was no reason to calculate upon, in a second trial. The plan, therefore, of dilating the urethra was adopted, and performed with Weiss' dilator with the most perfect success. The dilatation to the extent of an inch and half was completed without much pain in ten minutes; a pair of strong forceps were introduced, and the stone soon laid hold of. It appeared to be very large, and I found a good deal of resistance was to be offered to the extraction. The forceps was then very firmly grasped, in order that the hold might not be lost, when fortunately the stone gave way, and was reduced to many fragments, which were easily removed by the forceps, scoop, and repeated injections. A dose of tinct. opii was again given. No bad symptom followed, and far less pain was experienced, both during and after this operation, than the former. On the second day, the patient was able to retain her urine, and to void it with ease in ordinary quantity, which she has continued to do ever since.

"It would, perhaps, be unfair to draw any conclusion unfavourable to lithotrity, from a single, probably imperfect, and unsuccessful trial, at least on comparing it with lithotomy, which is always a hazardous operation; but in this instance it is perfectly legitimate to compare it with the operation of removing the stone by dilating the female urethra. A better opportunity could not have been found for forming a comparative estimate of the respective value of these different operations, and of showing the decided superiority of the latter over the former. Both operations were first attempts by the same operator, and therefore may be supposed equally unskilful, and both were performed on the same individual with a very short interval of time between them. If there was any difference, that difference was in favour of lithotrity, for at the commencement of dilatation the bladder was in a much more irritable state, than at the commencement of the former operation.

"Since the above, I have seen the operation of dilating the urethra performed by Dr. M'Farlane, on a girl three years and four months old. The dilatation to the extent of an inch was effected by the same dilator in ten minutes, and a stone the size of a pigeon's egg, extracted without difficulty. This girl was able to run about next day. Some incontinence of urine continued for a week or two, which has now gone off, and she is quite well."

There is this to be said in favour of, or against lithotrity, that as the management of the instruments for its performance requires more experience and tact than that of Weiss's dilator, so the odds were vastly in favour of the latter with a person equally unused to both. At the same time we must say that the balance of simplicity, celerity, and efficacy appears in this instance to have been on the side of dilatation.

---

